# Recovery from (treatment-resistant) depression after lifestyle changes and micronutrient precision supplementation: a preliminary field study in patients

**DOI:** 10.1186/s40359-023-01263-7

**Published:** 2023-08-11

**Authors:** Isabella M. Mader

**Affiliations:** Excellence Research, Vienna, Austria

**Keywords:** Depression, Nutrition, Precision nutrition, Laboratory tests, Cost containment

## Abstract

**Background and purpose:**

The incidence of depression is increasing, despite continued advances in psychological and pharmacological interventions. New treatment approaches are urgently needed. Here we assess the effects on depression of individualized micronutrient supplementation, in concert with a standard set of lifestyle changes.

**Methods:**

We conducted a small field-study with 17 participants in Austria. Patients with depression (*n* = 11) and healthy volunteers (*n* = 6) underwent laboratory serum analysis and filled out the DASS-21 and a questionnaire about their medical history and condition. The list of parameters to be tested in the serum analysis was derived from an expert heuristic compilation of factors known to influence depression, narrowed down to a workable list to be tested in this initial study. On the basis of the results, the participants (*n* = 17) received individualized recommendations for micronutrient supplementation, in collaboration with their treating physician. Participants followed the individual supplementation regime for two months, along with a standard set of lifestyle changes. After two months the laboratory serum analyses, the DASS-21, and the questionnaire were repeated.

**Results:**

All patients with micronutrient deficiencies were in the patient group; none of the healthy volunteers showed any micronutrient deficiencies. After two months of precision supplementation and lifestyle changes, all but one patient had recovered from their depression or had considerably improved. The one patient who didn’t recover was the only one with a known trigger of their depression (trauma). Of 11 patients with depression, the trigger was unknown for the other ten.

**Conclusions:**

These results have promising implications for further research, treatment, drug development, and public health. We propose that systematic screening of patients with symptoms of depression be developed for future research, medical care, and practice. Psychiatry and psychotherapy may see improved results once they no longer have to push against the underlying constraints of existing micronutrient deficiencies.

**Supplementary Information:**

The online version contains supplementary material available at 10.1186/s40359-023-01263-7.

## Introduction

Depression is one of the leading causes of disability [1], and its incidence has increased by at least 25% during the COVID-19 pandemic [2]. With a staggering 30% to 60% of patients resistant to current antidepressant therapies [3, 4] and 80% of patients lacking access to treatment worldwide [5], there is a dire need for new, affordable, simple diagnostic and therapeutic options.

Psychology and academic medicine have brought forth a wealth of insights into the many possible mechanisms underlying depression. Besides stressful life events and trauma, physiological and lifestyle factors can also trigger depression. In this field study, we evaluated the physiological and lifestyle factors that have been linked to depression and selected a subset of these to test in patients with depression. We used serum laboratory test results to recommend individualized micronutrient supplementation together with a standard set of lifestyle changes, and assessed the results of this intervention after two months.

### Nervous system function contributing to depression

Physiological factors contributing to depression include nervous system function, specifically neuromodulator metabolism and endocrine function, gut health, genetics, micronutrient deficiencies, and a series of comorbidities.

Some of these, especially nervous system function and neuromodulator metabolism form part of current teaching and practice regarding depression. Endocrine function, gut health, genetic factors, and micronutrient deficiencies have also been the subject of much research and are frequently observed in patients presenting with depression [6–11].

To identify the most promising factors for serum laboratory testing in our field study of patients, we scanned the literature for strong evidence. An expert heuristic evaluation was used to compile a list of factors for laboratory testing and for suggested lifestyle changes.

### Neuromodulator metabolism

Nervous system function and neuromodulator metabolism are one of the main backbones of the pathophysiology of depression. A core strain of existing and emerging treatments for depressive disorders target the monoamine neuromodulators dopamine and serotonin, in particular their synaptic availability. Although the effectiveness of antidepressant medication has been widely demonstrated, the detailed pathogenesis of depression remains unclear. Many patients’ symptoms remain resistant to current antidepressant treatment options.

### Neuromodulator metabolism

Fluctuations in neurotropic micronutrients can disrupt the central and peripheral nervous systems, some via their role as precursors and cofactors of neurotransmitter biosynthesis, some via their direct involvement in nervous system function. Mapping the essential precursors and cofactors of the dopamine–epinephrine and serotonin–melatonin biosynthesis pathways establishes the set of indicators to be considered, from which the expert heuristic departed. A more extensive set of indicators was developed to be offered for consideration in future research (see 4.2).

### Endocrine system

Endocrine system disorders, mainly regarding glucocorticoids and steroid hormones, have been identified as possible contributors to depression. A long history of academic medicine, clinical experience and preclinical investigation has implicated all major endocrine systems in the pathophysiology of depression.

### Lifestyle factors contributing to depression

We also looked at lifestyle changes with relevance to depression that can easily be incorporated into daily routines. After expert heuristic evaluation following the research detailed below, the lifestyle improvements selected for testing in the study focused on improving sleep quality, including a minimal amount of movement/sport, incorporating a fresh food diet (avoiding highly processed foods), and reducing of social isolation (if any).

### Social isolation

Social isolation is a known contributor to mental health conditions, although it is sometimes regarded as a "soft factor". The discovery that social isolation causes upregulation of neurokinin B (NkB) in the brain, leading to severe behavioral changes, provides metrics to support the consideration of social isolation in our study [12, 13].

Participants in this study were asked to meet at least one person per day; during lockdowns this was for a walk outside.

- Participants were asked in the questionnaire at the beginning whether they met people every day. In the questionnaire at the end of the study they were asked whether they incorporated the lifestyle change (if they lived in social isolation before) or if they could upkeep meeting others daily (results see 3.3).

### Lack of movement

Meta-analyses and systematic reviews have shown that exercise and physical activity are strongly associated with physical and mental health. Numerous studies point to exercise as an effective intervention in the treatment and prevention of depression [14, 15].

- Participants in our study were asked to practice a minimal amount of sport: 30 minutes of walking per day was sufficient.

### Sleep

Disordered sleep is one of the diagnostic criteria for depression and other mood disorders [16]. Dysregulation of the wake–sleep rhythm such that it is out of sync with the day–night cycle increases susceptibility to major depression, for example [17].

There was a significant increase in the prevalence of sleep disorders across all age groups during the COVID-19 pandemic, compared with pre-COVID-19 levels. Thomas et al. reported a higher prevalence of symptoms, although no aggravation of existing symptoms [18].

Dopamine and melatonin act as antagonists: Melatonin blocks dopamine release and dopamine reduces melatonin synthesis, each in response to light exposure [19]. Rahman et al. showed that secretion of melatonin is stopped abruptly by bright light and that intermittent light at night results in increased cortisol levels instead. Although it was previously thought that only very bright light could disrupt the melatonin and cortisol systems, more recent studies clearly show that even dim light from indoor illumination and electronic devices is sufficient to suppress melatonin secretion and considerably disrupt circadian cortisol rhythms [20].

- In the present study, participants were recommended to refrain from using artificial light from screens at night (television, computer screens, mobile devices).

### Diet/nutrition

Diet, nutrition, and gut health can promote or impair mental health in many ways. Here, only selected aspects with empirical support will be discussed. These include microbiome dysbiosis (disturbances of the intestinal flora), chronic autoimmune and inflammatory bowel diseases, and intestinal permeability, as well as the impairment of the catecholamine balance (dopamine, norepinephrine, epinephrine and their metabolites) by highly processed foods (cf. [21–23]).

### Highly processed foods

A recent meta-analysis of nutrient intake analyzed 14 representative national studies and found that the average consumption of highly processed foods varied widely among the countries studied. At the lower end of the scale were Colombia, Taiwan, and Brazil, with 15.9, 19.5, and 21.5 percent of highly processed foods in their diets. At the higher end of the scale were Canada, the United Kingdom, and the United States, with 47.7, 56.8, and 57.5 percent. The consequences of high consumption of processed food include not only a significantly higher calorie intake, but also a lower intake of micronutrients because highly processed foods are characterized by a dramatically lower nutrient content than natural and fresh foods [24]. Excessive sodium and insufficient potassium levels in the urine of adolescents were shown to be predictors of depression induced by malnutrition [25].

- Participants in the study were recommended to eat mostly fresh, natural, and organic foods and to refrain from eating highly processed foods wherever possible.

## Methods

### Overview and process

In this field study, we measured a subset of common monoamine biosynthesis and nervous system cofactors in 17 adults (11 patients with depression and 6 healthy volunteers) in Austria.

The study recruited both healthy volunteers and volunteers with depression through a single posting on Facebook. Volonteers messaged privately declaring interest. To participate in the study, individuals were required to be at least 18 years old. Participants with depression were required to have a diagnosis of depression but no other psychiatric conditions. To ensure transparency and informed consent, participants were provided with a complete information set about the study process, conditions, and data protection protocol. The data protection declaration contained the participant's name, while all other documents, including questionnaires and lab results, were labeled with a case number only. Participants provided written consent by signing the data protection declaration and agreeing to the study conditions.

Participant names were not entered into any database or digital files. Questionnaires were sent out as PDFs and returned by mail or email using the assigned case number. To further ensure the safety and well-being of the participants, all study recommendations were confirmed with their treating physicians.

The number of participants included in the study was determined based on the number of complete sets of two laboratory tests and two questionnaires that were returned. While some participants returned only one laboratory result or laboratory results containing only the standard blood count, a total of 17 participants successfully completed the entire study process and were included in the final analysis.

**Fig. 1:**
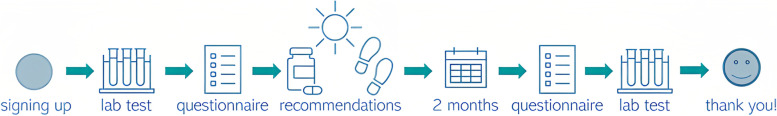
Study timeline (excerpt from the information provided to participants)

A laboratory serum analysis of a selection of parameters linked to depression was conducted, and on the basis of those results, participants were given an individualized micronutrient supplementation recommendation to be considered by their treating physician, along with standard lifestyle-change recommendations. The laboratory serum analysis was repeated two months later. To assess participants’ level of depression, the DASS-21 (Depression Anxiety and Stress Scale with 21 items) was used at the beginning and at the end of the study, together with general questions on current diagnoses, medication, and comorbidities. The timeline and process of the study is shown in Fig. 1.

#### Hypotheses

On the basis of current research, we proposed the following hypotheses:

- 1. Patients with depression are more likely to show micronutrient deficiencies than healthy controls.
- 2. Precision supplementation improves patients’ symptoms.
- 3. Adopting a set of healthy lifestyle habits supports relief or recovery.

### Parameter selection for the serum laboratory test

We tested a limited number of parameters related to depression in serum laboratory tests.

The following parameters were tested:

### Micronutrient deficiencies or insufficiencies

For this field study, a limited number of micronutrients should be tested. The following micronutrients were considered for testing in this field study, mainly because of their known or much researched involvement in neuromodulator metabolism or in comorbitities linked to depression.

All blood tests were analyzed in various licensed public laboratories in Austria (depending on the location of the participants that were spread out across the country).

#### Vitamin B9 (folate)

Vitamin B9 (folate) is required for the synthesis, repair, and methylation of DNA. Living cells need folate to divide and function. Folate deficiency typically results in anemia, irritability and behavioral disorders. Folate deficiency has been reported in depression as well [26]. A concrete connection of folate status with depression has been discussed for some time but is still considered unclear [27]. Leahy [28] shows that folate in its metabolized form L-methylfolate is a cofactor in the biosynthesis of serotonin, norepinephrine, and dopamine. However, when patients lack enzymes that convert folate to L-methylfolate, the contribution to the synthesis of the aforementioned neuromodulators cannot occur). Due to its connection with DNA methylation and neuromodulator metabolism as well as due to the link to insomnia and fatigue, folate was considered in the factors to be tested in this study.

Folate and vitamin B12 exhibit a codependency: Insufficiency of one can lead to insufficiency of the other [29]. Therefore, vitamin B12 was considered to be tested in this study as well.

Reference ranges considerably vary across countries.

Reference ranges used in the study: 3.9–19.8 mg/L [30].

#### Cobalamin (vitamin B12)

Cobalamin (vitamin B12) is a water-soluble vitamin that is consumed through diet and absorbed through the gastrointestinal tract. It is essential for neurological function, red blood cell formation, and DNA synthesis [31]. Deficiency of cobalamin can cause damage to peripheral nerves. In severe deficiency, patients can develop dementia and conditions such as psychosis with hallucinations, paranoia, or severe depression can occur [32].

Cobalamin (vitamin B12) is an essential cofactor in homocysteine metabolism. Homocysteine can be elevated in vitamin B12 deficiency or vitamin B12 uptake disorder and is considered a biomarker for cardiovascular disease and neurodegenerative diseases [32, 33].

In the remethylation of homocysteine, the methylated form of vitamin B12, methyl-cobalamin, is required in addition to folate [34]. Vitamin B12 is absorbed through the intestine, which is why diseases of the gastrointestinal tract can be triggers of insufficient cobalamin absorption. Therefore, cobalamin supplementation is provided as a standard after bariatric surgery because of the anatomic and functional changes of the intestine.

Reference ranges used in the study: 156–672 pmol/L [35].

#### Vitamin C (ascorbic acid)

Vitamin C is a water-soluble, essential vitamin that is mainly ingested with plant foods [36, 37]. The activated form of vitamin C is ascorbic acid. Vitamin C is an essential cofactor in the biosynthesis of dopamine, adrenaline, serotonin, and melatonin and therefore merits the inclusion in the laboratory testing in this study. The role of vitamin C in this is to maintain iron (Fe) in its reduced, divalent form (Fe + 2) [34]. The term vitamin C refers not to just one molecule, but to a family of five molecules with almost identical structure, differing only in their ionization and oxidative state. Only the reduced, ionized form of vitamin C, L-ascorbate, is responsible for the biological activity of the molecule [38].

A deficiency of vitamin C is associated with depression, anxiety, schizophrenia, and neuro-degenerative diseases such as Alzheimer's and Parkinson's [39].

BH4 is an essential cofactor in many enzymatic processes, including the biosynthesis of neurotransmitters [40]. BH4 levels in the body are maintained by vitamin C [37].

Due to its comprehensive influence on physical and mental health, its role as a cofactor in the biosynthesis of dopamine, adrenaline and serotonin and its essential role in iron metabolism vitamin C was included in the analysis parameters in this study, even though it is not usually a standard test in clinical care.

The shelf life of vitamin C in non-stabilized samples at 4 degrees Celsius is a maximum of 3 h [36]. Tubes coated with lithium heparin on the inside were used by the laboratories for vitamin C testing. Samples were stored and transported refrigerated, protected from light and uncentrifuged. Measurements are carried out by the laboratories using isocratic ultra-high performance liquid chromatography (UPLC).

Reference ranges used in the study: total vitamin C (ascorbic acid + dehydroascorbic acid) in plasma: 4–15 mg/L [36].

#### Vitamin D (ergocalciferol, cholecalciferol)

D vitamins are a group of sterols that have a hormone-like function. [34]. The dependence on sunlight and UV light respectively suggests that vitamin D deficiency may increase in times of lockdown if too little time is spent outdoors due to home office (for example, by not commuting to work). [40].

Vitamin D regulates the amount of levodopa synthesized in tyrosine hydroxylase [41], and acts as a rate-limiting cofactor in serotonin biosynthesis [42]. Vitamin D deficiency is among the most common vitamin deficiencies. [43].

Reference ranges used in the study: 25-hydroxy vitamin D (calcidiol): 20–120 mg/L (50–300 nmol/L). [44].

### Endocrine function

Endocrine system disorders, mainly regarding glucocorticoids and steroid hormones, have been identified as possible contributors to depression. A long history of academic medicine, clinical experience and preclinical investigation has implicated all major endocrine systems in the pathophysiology of depression. For this study, DHEA-S (dehydroepiandrosterone sulfate) (in serum) was selected for laboratory testing. To keep the laboratory investigation within the scope of this study within a feasible framework, only DHEA-S (dehydroepiandrosterone sulfate) (in serum) was selected from the group of neuroactive steroids as an indicator on stress and on the overall availability of neuro-active steroids.

Reference ranges for DHEA-S used in the study were: men 110–510 μg/dl, women: 15–325 μg/dl [45].

### Other tests

While some patients in our study presented with pre-existing comorbidities, specific diagnostic tests for these conditions were not conducted. Instead, participants were asked to provide information about their existing diagnoses as established by their treating physicians (see Sect. 2.4). To complement this self-reported data, a standard blood count was performed to generically cross-check and validate the information provided in the questionnaires.

### Questionnaire based on the DASS-21

The DASS-21 (Depression Anxiety Stress Scales with 21 items) is a set of three scales with seven items each, for participants to self-report and for clinicians to measure the emotional states of depression, anxiety, and stress.

The depression scale evaluates a range of negative emotions, including dysphoria, hopelessness, devaluation of life, self-deprecation, lack of interest or engagement, anhedonia, and inertia. The anxiety scale evaluates various physiological and psychological symptoms of anxiety, such as autonomic arousal, skeletal muscle effects, situational anxiety, and the subjective experience of anxious affect. The stress scale is particularly responsive to chronic, nonspecific arousal levels, and assesses difficulties with relaxation, nervous arousal, as well as tendencies to become easily upset or agitated, irritable, over-reactive, or impatient.

In the DASS-21, somatic items are avoided, and the questions focus instead on the core psychological aspects of depression, anxiety, and stress. In classification of clinical cases, the DASS-21 shows better sensitivity and specificity than the HADS (Hospital Anxiety and Depression Scale) [46, 47].

In this study, the DASS-21 was used as a starting point to generate quantitative data and to classify participants in terms of their symptoms.

The questionnaires used at the beginning and at the end of the study are available as supplementary files 1 and 2.

### DASS-21 questionnaire

Participants were asked to read each statement and circle a number 0, 1, 2 or 3 which indicates how much the statement applied to them over the past week. There were no right or wrong answers. They were asked not to spend too much time on any statement.

The standard rating scale was provided as follows:

- 0 Did not apply to me at all.
- 1 Applied to me to some degree, or some of the time.
- 2 Applied to me to a considerable degree or a good part of time.
- 3 Applied to me very much or most of the time.

The standard DASS-21 questions were:

- 1. I found it difficult to calm down.
- 2. I felt that my mouth was dry.
- 3. I couldn't experience any positive emotions at all.
- 4. I had breathing problems (e.g. excessively fast breathing, shortness of breath without physical exertion).
- 5. It was difficult for me to motivate myself to get things done.
- 6. I tended to overreact to situations.
- 7. I trembled (e.g. in my hands).
- 8. I found everything exhausting.
- 9. I worried about situations in which I could panic and make a fool of myself.
- 10. I felt like I couldn't look forward to anything anymore.
- 11. I noticed that I became easily agitated.
- 12. I found it difficult to relax.
- 13. I felt down and sad.
- 14. I reacted angrily to anything that prevented me from continuing my current activity.
- 15. I felt close to a panic attack.
- 16. I was unable to get excited about anything.
- 17. I didn't feel like I was worth much as a person.
- 18. I found myself quite sensitive.
- 19. I felt my heartbeat without having physically exerted myself (e.g. feeling of palpitations or skipped beats).
- 20. I felt anxious for no reason.
- 21. I felt that life was meaningless.

Scoring of the DASS-21 (not included in the patient version):

- The depression scale (D) consists of items 3, 5, 10, 13, 16, 17, and 21.
- The anxiety scale (A) includes questions 2, 4, 7, 9, 15, 19, and 20.
- The stress scale (S) comprises items 1, 6, 8, 11, 12, 14, and 18.

Total scores for depression, anxiety, and stress are obtained by summing the relevant item scores. Total score per scale (D, A, and S) are subsequently multiplied by 2.

Table 3 contains the standard DASS-21 severity levels and the color code that is also used in Table 2 showing the individual results.

### Additional questions in questionnaire 1 (see supplementary materials no. 1)

We extended the DASS-21 questionnaire with additional questions about living conditions, working conditions, existing diagnoses, medication, known triggers of depression, and allergies as well as current lifestyle (sport, diet, social isolation, or social stress).

- 22. I am currently undergoing treatment for the following diagnoses.
- 23. I am currently taking the following medications and/or supplements.
- 24. I have the following allergies/intolerances.
- 25. If you are currently not feeling well, please briefly describe in your own words how this manifests.
- 26. How long have you been feeling this way? (only if you are currently not feeling well, otherwise please continue with question 28)
- 27. Was there a trigger? (only if you are currently not feeling well, otherwise please continue with question 28)
- 28. Do you have any general medical conditions, especially such as diabetes, high blood pressure, etc.?
- 29. What are you currently doing for your well-being? Exercise? Special diet? Something else? Since when? What works well?
- 30. Do you meet many or few people in your daily life - professionally or privately? How was that (different?) under Covid? Less/more stress? Less pleasant? Or more pleasant?
- 31. What else do you think is important information—or what else would you like to tell us?

### Additional questions in questionnaire 2 (see supplementary materials no. 2)

The additional questions were slightly adapted to learn about the progress made or any other developments and outcomes.

- 22. Please describe briefly in your own words how you are currently feeling.
- 23. Have there been any changes in other diagnoses? (if applicable).
- 24. In the last 2 months, I have taken the recommended vitamins (check to select).
  ☐ consistently
  ☐ modified: (how?)
  ☐ stopped: (why?)
  ☐ other: (please specify if possible)
- 25. In the last 2 months, I have followed the following behavioral recommendations
  ☐ socialized more
  ☐ exercise (please tell us what type): _
  ☐ avoided bright light/phone/TV at night
  ☐ ate fresh foods, avoided heavily processed foods
  How successful was it? ____
  Comments:
- 26. Which micronutrients/vitamins and behavioral recommendations will you continue to follow?
- 27. Were the study materials useful, understandable, and interesting?
  If you would like to provide feedback, we would be very grateful.
- 28. May we contact you again in a few months to ask how you are doing?
- 29. What else do you think is important information to provide or anything else you would like to tell us?

## Recommendations for participants

### Precision supplementation

Following the participants' lab test results, they received personalized recommendations for micronutrients that catered to their specific needs (if any). They were advised to discuss these recommendations with their treating physician and take the suggested micronutrients for a minimum of 2 months. In case they wished to discontinue, it would not pose any issues.

Supplementation was recommended for patients with deficiency or insufficiency in vitamin B9 (folate), vitamin C and vitamin D. There was no case with vitamin B12 deficiency or insufficiency. For folate the methylated preparation was recommended to bypass any potential methylation-related polymorphisms (e.g. MTHFR) [48, 49].

Patients were encouraged to contact us or their treating physician with any queries or apprehensions that may arise.

### Lifestyle recommendations

All patients were provided with a standard set of lifestyle recommendations that were derived from Sect. 1.2.

### Social isolation

Participants were recommended to meet other people daily. During lockdowns, even meeting with distance was important for health, both mental and physical. Participants were informed that people in social isolation can develop unfavorable body chemistry after a short period of time. A walk with a friend or sitting an hour in a café were sufficient for the purposes of the study.

### Physical movement/sport

Participants were advised to incorporate a minimum of 30 minutes of physical activity per day as it has been shown to contribute significantly to the amelioration of depressive symptoms. Supplementary information was provided to underscore the positive impact of physical activity on various bodily functions, including the hormone system, blood circulation, and oxygen supply, among others. Depending on the participant's current fitness level, even a brief half-hour stroll outdoors could prove efficacious in promoting mental well-being.

### Sleep quality

Participants were asked to abstain from exposure to bright lights at night, including electronic devices such as mobile phones and televisions. Instead, they were encouraged to safely expose themselves to natural daylight and allocate some time outside every day. The information read that this was particularly important to bear in mind when working remotely, where this was often overlooked. Participants were apprised of the fact that bright lights are perceived as a waking signal, which can impede sleep quality, potentially leading to insomnia and compromising their overall health and performance. Moreover, that such exposure can have an adverse impact on their energy levels and mood.

### Diet/nutrition

Participants were advised to opt for a diet rich in fresh produce and to avoid highly processed foods. Furthermore, participants were informed about the well-established link between depression and gut health, and about the potential adverse effect of intestinal dysbiosis on mental health. While gut health issues may not constitute the sole cause of depression, participants were advised to switch to a nutrient-dense diet consisting of fresh ingredients. In case of any uncertainty, participants were encouraged to seek medical advice from their treating physician regarding recommendations on individual dietary choices.

## Results

In our small field study, 11 of the 17 participants presented with varying degrees of depression. The healthy group comprised the remaining six participants. The age distribution was between 18 and 68 years, and 4 were males, 13 were females. Two patients were taking SSRIs (selective serotonin reuptake inhibitors) (see Table 1 for more details).

**Table 1:** Overview of the results

**Case no**	**m/f**	**vit. start** 1 = deficient	**vit. end**	**DHEA-S start**	**DHEA-S end**	**DASS-21 start**	**DASS-21 end**	**DASS-21 diff**	**suppl. start** 1 = yes 0 = no	**wrong suppl. start** 1 = yes	**diagnosis at start**	**depr. duration**	**depr. at start** 1 = yes 0 = no	**Status end (free text)**	**existing antidepr. med**	**additional info/ background/trigger**
1	F	1	0	1	1	28	4	-24	1	1	depression	6 mon	1	very good	0	
2	F	0	0	0	0	52	60	+ 8	1		depression	6 mon	1	unchanged	0	trauma
3	F	0	0	0	0	20	58	+ 38	1				0	life incident	0	
4	F	0	0	0	0	22	28	+ 6	1				0	stress increased	0	Hashimoto
5	F	1	0	0	0	68	36	-32	1	1	depression	4 mon	1	good	1	injury
6	F	1	0	0	0	48	40	-8	0		depression	5 + yrs	1	better	1	SSRI
7	F	0	0	0	0	32	28	-4	1		depression	5 + yrs	1	much better	0	Hashimoto
8	F	0	0	0	0	2	2	0	1				0	unchanged	0	
9	F	1	0	0	0	74	14	-60	0		depression	2 mon	1	very good	0	
10	F	1	0	1	0	64	28	-36	1	1	depression	6 mon	1	much better	0	Hashimoto
11	M	0	0	0	0	52	22	-30	1				0	better	0	
12	F	1	0	0	0	86	28	-58	0		depression	2–5 yrs	1	good	1	SSRI
13	M	0	0	0	0	8	6	-2	1				0	very good	0	
14	F	1	0	0	0	50	12	-38	1	1	depression	5 + yrs	1	good	0	
15	M	1	0	0	0	32	0	-32	1	1	depr.episodes	6 mon	1	very good	0	
16	M	1	0	0	0	44	8	-36	0		depr.episodes	2–5 yrs	1	good	0	
17	F	0	0	0	0	8	2	-6	1				0	very good	0	

abbr.: *m* male, *f* female, *vit* vitamins B9/B12/C/D, *DASS-21* Depression-Anxiety-Stress-Scales with 21 items, *suppl.* supplementation, diagnosis at start = diagnosed (by treating physician), *antidepr*. antidepressant, *add*. additional, *OCD* obsessive compulsive disorder, *SSRI* selective serotonin reuptake inhibitor

Not all the parameters could be determined for all participants. Furthermore, because of the small number of participants, we cannot draw any general conclusions. Nevertheless, an interesting picture emerged from our results:

- All individuals with micronutrient deficiency or insufficiency (*n* = 9) belonged to the group with depression (*n* = 11). The two patients without deficiencies were already being supplemented at the beginning of the study.
- None of the healthy subjects (*n* = 6) presented with micronutrient deficiency or insufficiency.
- At the beginning of the study, four patients in the depression group were not taking any supplements and five patients were supplementing inadequately.
- After two months of adherence to precision supplementation and behavioral recommendations, 10 of 11 participants in the patient group reported an improvement of or recovery from their depression.
- A single patient (case number 2) remained unchanged in terms of how she felt. This was the only participant whose depression was trauma-induced.

During the two months of the study, one of the participants (case number 3) from the healthy participant group developed a depressive episode. She did not show any micronutrient deficiency or insufficiency (at least with respect to the parameters examined here) and had used nutrition supplementation before the beginning of the study. She attributed her condition to a crisis in the family (a child fell severely ill). She (and the child) had recovered at the time of writing this report.

One patient’s depression was trauma-induced, and another patient reported that their depression started after a sports injury. The other nine patients reported that the reason for their depression was unknown. The only patient who did not recover or improve was the one whose depression was trauma-induced: this patient was already taking supplements and adhering to the suggested lifestyle changes before the start of this study.

Two patients with depression were already taking supplements at the beginning of the study and did not show any micronutrient insufficiency. One of these patients achieved a significant improvement in symptoms by adhering to the behavioral recommendations during the two months of the study.

At the end of the two-month study period, none of the participants showed micronutrient insufficiencies/deficiencies in their laboratory results.

### Interpretation of the laboratory results

Of the nine participants who were found to have micronutrient deficiencies, eight were treated with precision supplementation and reported improvement or recovery at the end of the two-month treatment period. For one participant, correction of vitamin C deficiency was not indicated due to an existing autoimmune condition. None of the healthy participants showed micronutrient deficiency or insufficiency.

Despite the small group of participants, two conclusions can be drawn: Laboratory analysis of micronutrients appears to be indicated in patients with depression, ideally before other treatment is considered. However, the options for supplementation are limited in patients on existing antidepressant medication because of known interactions (especially with amino acids or n-3 fatty acids) [50, 51]. We report the following individual results:

- Vitamin B9/Folate: One patient showed folate deficiency.
- Vitamin B12: All participants had levels within the normal range.
- Vitamin C: Values were available for only seven of the 17 participants. Of these, two participants had low values for vitamin C. One of these participants had a long history of autoimmune disease (Hashimoto's) and vitamin C administration was not indicated. Improvement in this participant was achieved via lifestyle changes.
- Vitamin D: Nine of 11 patients with depression had vitamin D deficiency, the other two patients were on vitamin D supplementation. This result is consistent with international findings that vitamin D deficiency is widespread worldwide. Approximately 40 percent of individuals in Europe have vitamin D insufficiency or deficiency, with 13 percent having a severe deficiency [43].

### Interpretation of survey results

Out of the 17 participants, 11 exhibited different levels of depression. The remaining six belonged to the healthy group. The duration of depression in the patient group ranged from 2 months to 28 years. The ages of the participants varied between 18 and 68 years (the median age being 45), with 4 being male and 13 being female.

Survey results from the DASS-21 inventory confirmed each of the reported diagnoses, both at the start and at the end of the study. Depression severity levels according to the DASS-21 varied between mild (one case) and very severe depression (three cases). There was one case with severe depression and another seven cases with moderate depression at the start of the study.

Onset of depression of patients (participants with depression): For six of the 11 patients, their depression began during the second COVID-19 related lockdown in Austria, between November 2020 and February 2021. Another two patients had been in treatment for depression for between 2 and 5 years. The remaining three patients had been in treatment for depression for more than 5 years.

The DASS-21 ratings of the ten patients that improved were in the beginning at an average 52.6 points (ranging between 28 for mild depression and 86 points for very severe depression according to the rating scale). At the end of the study their average rating had come down to 19.8 points (minus an average of 32.8 points). One patient did not improve (the only trauma induced depression patient); her rating went up by 8.0 points during the study (from 52 to 60 points, both in the range of moderate depression).

Table 2 shows the duration of illness at the start of the study and the progress made by the end of the study. Table 3 provides the severity levels used in Table 2.

**Table 2:** DASS-21 results in detail

Participants	1A	1B	2A	2B	3A	3B	4A	4B	5A	5B	6A	6B	7A	7B	8A	8B	9A	9B	10A	10B	11A	11B	12A	12B	13A	13B	14A	14B	15A	15B	16A	16B	17A	17B
depression (D) value	**10**	**0**	**20**	**18**	**6**	**26**	**6**	**4**	**34**	**14**	**20**	**14**	**14**	**12**	**2**	**2**	**32**	**2**	**22**	**10**	**18**	**6**	**28**	**10**	**0**	**0**	**14**	**2**	**8**	**0**	**16**	**0**	**0**	**0**
Severity	**1**	**0**	**2**	**2**	**0**	**3**	**0**	**0**	**4**	**2**	**2**	**2**	**2**	**1**	**0**	**0**	**4**	**0**	**3**	**1**	**2**	**0**	**4**	**0**	**0**	**0**	**2**	**0**	**0**	**0**	**2**	**0**	**0**	**0**
anxiety (A) value	**4**	**0**	**12**	**14**	**2**	**8**	**8**	**12**	**8**	**0**	**14**	**12**	**0**	**0**	**0**	**0**	**12**	**0**	**16**	**4**	**10**	**6**	**18**	**4**	**4**	**2**	**14**	**0**	**2**	**0**	**8**	**0**	**0**	**0**
Severity	**0**	**0**	**2**	**2**	**0**	**1**	**1**	**1**	**1**	**0**	**2**	**2**	**0**	**0**	**0**	**0**	**2**	**0**	**3**	**0**	**2**	**0**	**3**	**0**	**0**	**0**	**2**	**0**	**0**	**0**	**1**	**0**	**0**	**0**
stress (S) value	**14**	**4**	**20**	**28**	**12**	**24**	**8**	**12**	**26**	**22**	**14**	**14**	**18**	**16**	**0**	**0**	**30**	**12**	**26**	**14**	**24**	**10**	**40**	**14**	**4**	**4**	**22**	**10**	**22**	**0**	**20**	**8**	**8**	**2**
Severity	**0**	**0**	**2**	**3**	**0**	**2**	**0**	**0**	**3**	**2**	**0**	**0**	**1**	**1**	**0**	**0**	**3**	**0**	**3**	**0**	**2**	**0**	**4**	**0**	**0**	**0**	**2**	**0**	**2**	**0**	**2**	**0**	**0**	**0**
Total	**28**	**4**	**52**	**60**	**20**	**58**	**22**	**28**	**68**	**36**	**48**	**40**	**32**	**28**	**2**	**2**	**74**	**14**	**64**	**28**	**52**	**22**	**86**	**28**	**8**	**6**	**50**	**12**	**32**	**0**	**44**	**8**	**8**	**2**

*1A, 2A, …* = *patient number at the beginning of the study, 1B, 2B, …* = *patient number at the end of the study. Severity see *Table 3

**Table 3:** DASS-21 severity levels and color code used in Table 2

		D	A	S
normal	0	0–9	0–7	0–14
mild	1	10–13	8–9	15–18
moderate	2	14–20	10–14	19–25
severe	3	21–27	15–19	26–33
very severe	4	28 +	20 +	34 +

### Lifestyle interventions

Avoiding television and mobile phone use for at least half an hour before going to bed was the least adopted behavioral change: five of 11 patients did not implement this change, although six did. On the other hand, making the effort to meet people at least once a day—even for an outside walk during lockdowns—was incorporated by all participants (Table 4).

**Table 4:** Patients’ adherence to lifestyle protocols

depressed patients with asterisk	1*	2*	**3**	**4**	5*	6*	7*	**8**	9*	10*	**11**	12*	**13**	14*	15*	16*	**17**
antidepressant medication	NO	NO	NO	NO	NO	YES	NO	NO	NO	NO	NO	YES	NO	NO	NO	NO	NO
Depression since	6 months	6 months	n.a	n.a	4 months	5 + years	5 + years	n.a	2 months	6 months	n.a	2–5 years	n.a	5 + years	6 months	2–5 years	n.a
activities before the start of the study	sport	sport healthy diet	sport	sport medi-tation	none	sport	sport	sport healthy diet	sport medita-tion	sport	none	none	healthy diet	healthy diet	sport	sport	healthy diet
social isolation during Covid-19 lockdowns	YES	NO	YES	NO	YES	NO	YES	NO	NO	YES	NO	NO	NO	NO	NO	NO	NO
individualized supplements taken as specified	YES	YES	YES	YES	YES	YES	YES	YES	YES	YES	YES	YES	YES	YES	YES	YES	YES
met at least 1 person daily	YES	YES	YES	YES	YES	YES	YES	YES	YES	YES	YES	YES	YES	YES	YES	YES	YES
sport/movement 30 min daily	YES	YES	YES	YES	YES	YES	YES	n.s	YES	YES	JA	NO	YES	YES	YES	YES	NO
avoid mobile/TV before bed/at night	YES	YES	NO	YES	none	NO	YES	n.s	YES	YES	NO	NO	YES	YES	NO	NO	YES
healthy diet	YES	YES	YES	YES	NO	NO	YES	n.s	YES	YES	YES	YES	YES	YES	YES	NO	YES
condition at the end of the study period	very good	unchanged	worse	good	good	better	good	good	good	good	good	good	good	good	very good	good	good

*n.s. not specified, n.a. not applicable*

* patients diagnosed with depression at the beginning of the study

For one patient with vitamin C deficiency supplementation was not considered because of an existing autoimmune condition. She reported that adopting the recommended set of lifestyle changes for the two-month study period contributed to improving her depression.

### Hypothesis verification

#### Hypothesis 1: Patients with depression are more likely to show micronutrient deficiencies than healthy controls

The clarity of the results was striking: 9 of 11 patients showed deficiencies, but none of the healthy controls did, while the 2 patients without deficiencies were already supplementing at the beginning of the study.

#### Hypothesis 2: Precision supplementation improves patients’ symptoms

All but one micronutrient-deficient patient reported an improvement in their condition or recovery from depression. The only patient who did not improve or recover was the only patient with trauma-induced depression who was already receiving extensive supplementation at the start of the study.

#### Hypothesis 3: Adopting a set of healthy lifestyle habits supports relief or recovery

Although the recommended lifestyle changes were adopted by most patients (unless they were already following the recommendations at the start of the study) a separate contribution to well-being cannot be tracked for these changes. However, one patient with micronutrient deficiency in vitamin C that could not be corrected because of an existing autoimmune condition reported that adopting the recommended set of lifestyle changes for the two-month study period contributed to improving her depression.

## Discussion

This study shows that targeted serum laboratory tests and precision supplementation may help improve depression or lead to recovery. Given the strong body of research on the individual factors tested, this result is not too surprising. However, the proportion of patients who recovered or improved was high (10 of 11), and even patients with longer-term treatment-resistant depression showed improvements. It is also noteworthy that all participants with micronutrient deficiencies belonged to the patient group; none of the healthy controls showed any marked deficiencies, despite the small size of the group.

Of the nine patients who reported that the cause or trigger of their depression was unknown, eight had micronutrient deficiencies and recovered or improved after precision supplementation. This raises a new research question: Could a high proportion of patients with depression of unknown cause have micronutrient-deficiency-induced depression? If our results generalize even slightly, this would open up a promising avenue of treatment.

### Precision medicine/precision nutrition requires laboratory results

Supplementation without prior laboratory testing is unlikely to achieve an adequate level of accuracy. The results from this small field study are promising and, we argue, merit the inclusion of a laboratory panel for all patients presenting with depression. Indeed, the main take-away from this study may be that laboratory screenings should play an important role before any type of treatment for depression is considered.

Depending on the clinical assessment of the particular patient, the list of parameters of relevance in the pathophysiology of depression listed above (Table 4) may be a useful starting point for examination.

### Mapping depression: Parameters of relevance in the pathophysiology of depression

After analyzing the recent advances in academic medicine on physiological factors and comorbidities that may contribute to depression, the below list of potential test parameters emerged (Table 5).

**Table 5:** Summary of potential testing parameters established via expert heuristic evaluation

Value	Relevance regarding depression / clinical presentation
Thiamine (vitamin B1)	In thiamine deficiency, the activity of the oxidative decarboxylation of pyruvate and α-ketoglutarate is decreased, resulting in impaired cellular function because of decreased production of ATP (adenosine triphosphate) [34]
Riboflavin (vitamin B2)	Riboflavin is involved in tryptophan and iron metabolism [10, 52]. Deficiency is rare
Niacin (vitamin B3)	Essential cofactor of dopamine and serotonin biosynthesis (Figs. 2 and 3)
Pantothenic acid (vitamin B5)	Involved in the development of the CNS and in the formation of acetylcholine, which is linked to depression [10]. Deficiency is rare
Pyridoxine (vitamin B6)	Essential cofactor of dopamine and serotonin biosynthesis (Figs. 2 and 3)
Biotin (vitamin B7)	Essential enzyme cofactor in the metabolism of fats and amino acids [53]. Deficiency is rare
Folate (vitamin B9)	Essential cofactor of dopamine and serotonin biosynthesis as a precursor of SAMe (see 2.3.1) (Figs. 2 and 3)
Cobalamin (vitamin B12)	Vitamin B12 deficiency is a common cause of various neuropsychiatric symptoms [31]. Higher levels correlate with improved depression treatment outcomes [10]
Ascorbic Acid (vitamin C)	Essential cofactor of dopamine, adrenaline (Fig. 2) and serotonin (Fig. 3) biosynthesis and essential in iron metabolism (see 2.3.1)
Ergocalciferol, cholecalciferol (vitamin D)	Essential cofactor of dopamine and serotonin biosynthesis. One of the most prevalent vitamin deficiencies (see 2.3.1)
Iron (Fe)	Essential cofactor of dopamine and serotonin biosynthesis (Fig. 3)
Magnesium (Mg)	Essential cofactor of adrenaline biosynthesis (Fig. 2)
Copper (Cu)	Essential cofactor of adrenaline biosynthesis (Fig. 2)
Zinc (Zn)	Essential for methylation [54]. Deficiency is common in patients with depression [55]
Potassium (K)	Essential for the proper functioning of the nervous system
Phosphate (PHOS)	Will be elevated when vitamin D is overdosed [56]
Dopamine	Classic target of some antidepressant medications. Not usually tested
Serotonin	Classic target of some antidepressant medications. Not usually tested
Oxytocin	Involved in biological processes that are altered in patients with depression. Mechanisms involved with depression part of ongoing research [57]. Not usually tested
β-endorphin	A growing body of evidence points to a strong link between opioid system dysfunction and psychopathology [58]
SAMe (S-Adenosyl-methionine)	Essential for methylation, e.g., in the metabolism of folate and cobalamin metabolism. Essential cofactor for biosynthesis of adrenaline and melatonin (Figs. 2 and 3)
PUFAs: fatty acids Omega 3/n-3 EPA, DHA	Essential cofactor of dopamine and serotonin biosynthesis (see Figs. 2 and 3). Typically analyzed together with Omega 6/n-6. Varying standards for testing [42]
(essential) amino acids	Essential cofactors of dopamine and serotonin biosynthesis and profound impact on neurotransmitter synthesis and metabolism (Figs. 2 and 3)
DHEA-S (+ cortisol)	Most abundant steroid hormone in the circulation. Converted to sex steroid hormones. Important indicator of hormone health
Sex steroid hormones: E2, E1, P4, TTBF	Significant influence on brain physiology, modulating and stimulating effect on neuronal activity in cognition, behavior and agonistic or antagonistic effects toward various neurotransmitters
Thyroid panel: TSH, T4, T3, rT3 + autoimmune conditions	Thyroid conditions are frequently associated with depression. Hypothyroidism is considered a cause of or strong risk factor for depression, mainly in women
PRL	Prolactin (PRL) is regulated by dopamine in the brain, which makes it a possible indicator of dopamine function
HGH, IGF-1	Promising research links deficiency in HGH and its product IGF-1 to depression
Homocysteine	Indicator of methylation-related polymorphisms (e.g. MTHFR) (48, 49)
CRP	Indicator of inflammatory comorbidities that can trigger or aggravate depression
Standard blood count	Standard starting point for diagnosis

The parameters in Table 5 include testing for comorbidities known to trigger or contribute to depression, and micronutrients necessary for nervous system function including neuromodulator metabolism: fluctuations in neurotropic micronutrients can disrupt the central and peripheral nervous systems, some via their role as precursors and cofactors of neurotransmitter biosynthesis, some via their direct involvement in nervous system function. Mapping the essential precursors and cofactors of the dopamine–epinephrine and serotonin–melatonin biosynthesis pathways establishes a generic set of indicators to be considered.

**Fig. 2:**
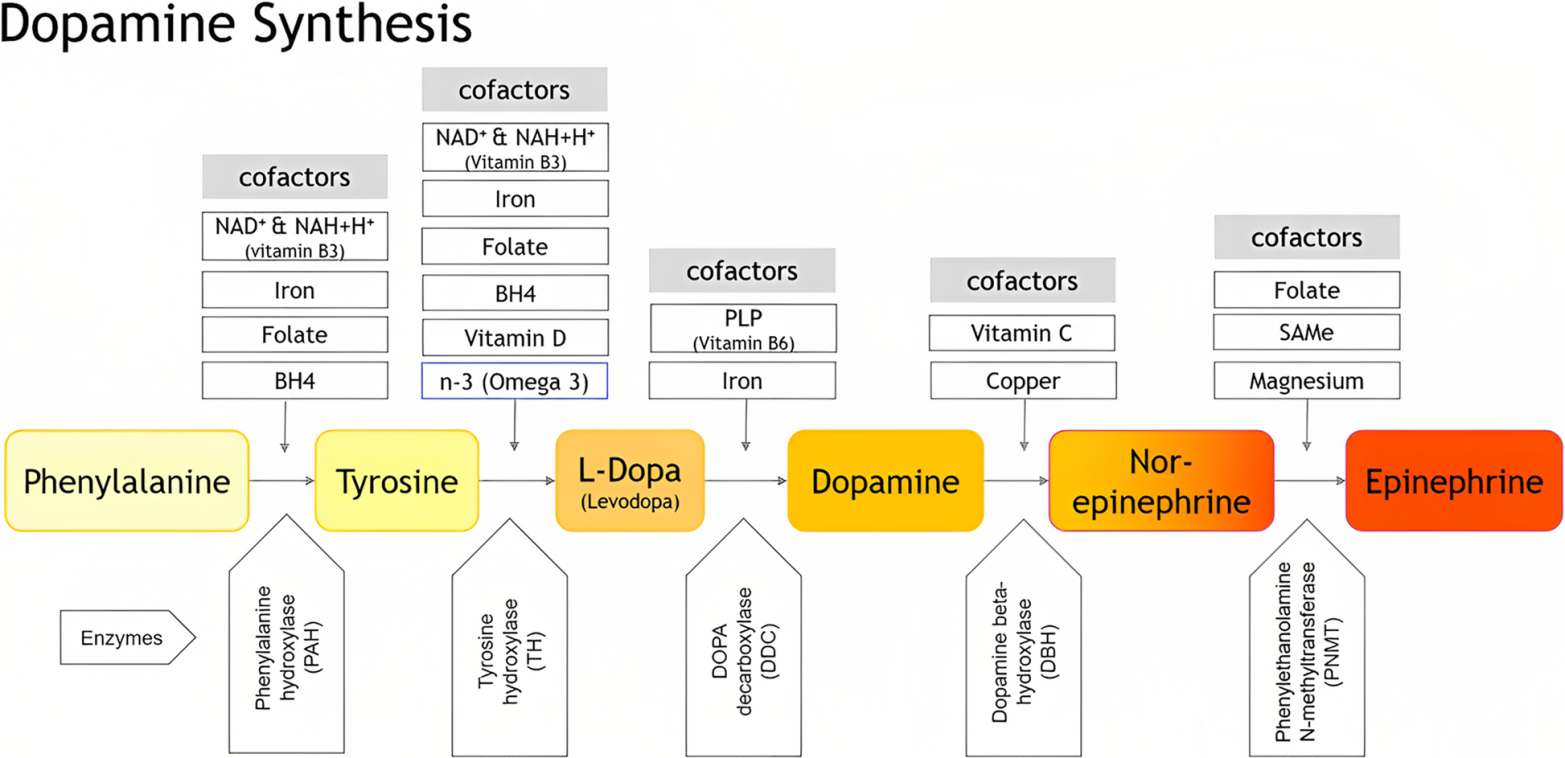
The dopamine synthesis pathway and its cofactors

Dopamine biosynthesis has phenylalanine and tyrosine as precursors and uses the rate-limiting cofactors vitamin B3, iron (Fe), folate, tetrahydrobiopterin (BH4), vitamin D, and vitamin B6 to complete its synthesis. The synthesis of epinephrine from dopamine requires the cofactors vitamin C, copper (Cu), folate, S-adenosyl methionine (SAMe), and magnesium (Mg) [28, 59, 60] (Fig. 2).

**Fig. 3:**
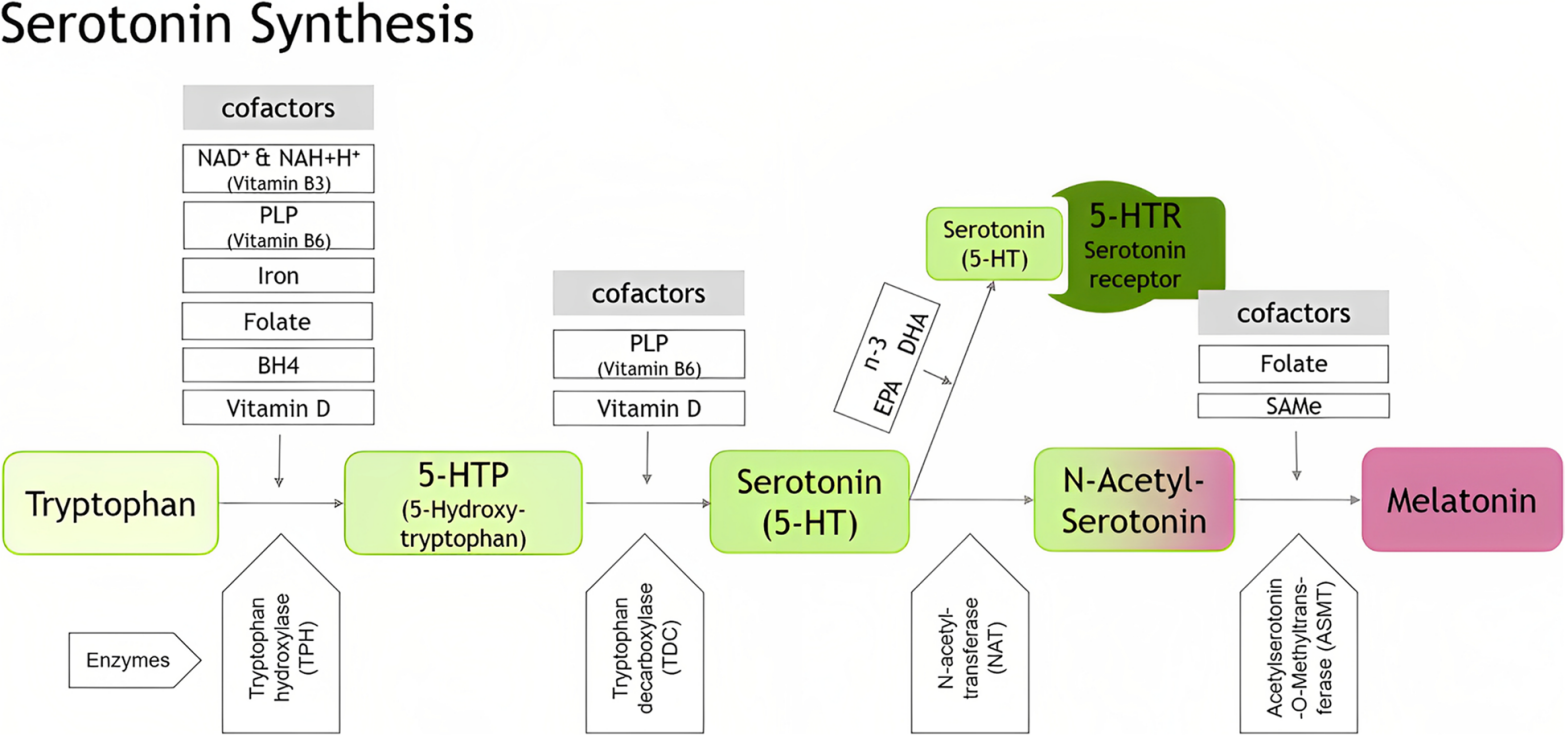
The serotonin synthesis pathway and its cofactors

Serotonin (Fig. 3) is synthesized from tryptophan and requires the cofactors vitamins B3, vitamin B6, iron, folate, tetrahydrobiopterin (BH4), and vitamin D [61, 62] (Fig. 3). Serotonin release is influenced by eicosapentaenoic acid (EPA), and serotonin receptor function is influenced by docosahexaenoic acid (DHA), both components of the omega-3 polyunsaturated fatty acid complex (PUFA) [42]. Melatonin is synthesized from serotonin via the cofactors folate and SAMe [62].

### Potential for antidepressant drug development

The role of micronutrients and individualized/precision supplementation opens novel and largely untapped routes for drug development in the supplements space currently claimed by the food industry (the recent acquisition of Bountiful Company by Nestlé makes the latter the world’s biggest supplier of supplements). The pharmaceutical industry might be better suited to serve what may become a quickly growing need in the clinical field.

A number of micronutrient-based pharmaceutical preparations are already available, but the large and growing body of evidence in the field seems likely to open up the potential for drug development. Growing interest across patient groups will likely rapidly expand the market for micronutrient-based pharmaceutical preparations.

### Potential for medical practice

Supplements are generally safe but are best taken in consultation with a physician and taking into account existing medication and conditions. This could potentially open up a new line of treatments in medical practice, as more patients seek care and as indicated by the rapidly growing supplements market. New patient groups who will not consider taking legacy antidepressants may decide to embark on precision supplementation, and this may hold true for prevention as well as treatment.

### Potential for psychotherapy

Patients with micronutrient deficiencies tend to present with various health conditions, of which depression is only one. Psychotherapy, irrespective of the type, cannot by its very nature remediate a concrete physical deficiency. Therapies that address the physiological level of micronutrient deficiencies could be used in concert with psychotherapy to significantly increase the chances of recovery and the success of the psychotherapy.

## Conclusion

Serum laboratory tests selected on the basis of existing knowledge of the role of micronutrients in depression may be a promising additional option for treatment and may lead to novel drug development. Furthermore, reduced therapy resistance during psychiatric treatments may occur once underlying micronutrient deficiencies are treated. We may therefore observe improved results from psychotherapy once it no longer has to push against the underlying constraints of existing micronutrient deficiencies.

Given the promising results of this pilot study, further investigations, including a broader range of parameters (4.2.), seem merited.

### Supplementary Information

**Additional file 1.** 

**Additional file 2.**

## Data Availability

The data analyzed during the study are not publicly available due to the fact that individual participants in the study are interrelated (e.g. mother and daughter) and publication could leak medical data. The data relevant for the study are provided within the article. Further data are available from the corresponding author on reasonable request.

## Abbreviations

ATP: Adenosine triphosphate
BH4: Tetrahydrobiopterin
CRP: C-reactive protein
Cu: Copper
DASS-21: Depression Anxiety and Stress Scale with 21 items
DHEA-S: Dehydroepiandrosterone sulfate
DHA: Docosahexaenoic acid
DM: Diabetes mellitius
E1: Estrone
E2: Estradiol
EPA: Eicosapentaenoic acid
Fe: Iron
GBD: Global Burden of Disease
HADS: Hospital Anxiety and Depression Scale
HGH: Human growth hormone
HPA: Hypothalamic–pituitary–adrenal axis
HPT: Hypothalamic–pituitary–thyroid axis
HPG: Hypothalamic–pituitary–gonadal axis
HPP: Hypothalamic–pituitary–prolactin axis
HPS: Hypothalamic–pituitary–somatotropic axis
HS CRP: High-sensitivity C-reactive protein
IBD: Inflammatory bowel disease
IGF-1: Insulin-like growth factor 1
K: Potassium
Mg: Magnesium
MTHFR: Methylenetetrahydrofolate reductase
n-3: Omega 3 fatty acids
n-6: Omega 6 fatty acids
NIH: National Institutes of Health
NkB: Neurokinin B
PHOS: Phosphate
P4: Progesterone
PRL: Prolactin
PUFA: Polyunsaturated fatty acid
SAMe: S-adenosyl methionine
SSRI: Selective serotonin reuptake inhibitor
TTFB: Testosterone panel: testosterone, total, free, bioavailable, serum
Zn: Zinc
