# Supplementary material for: Recovery from (treatment-resistant) depression after lifestyle changes and micronutrient precision supplementation: a preliminary field study in patients
Source: BMC Psychol. 2023 Aug 11;11:229. doi: 10.1186/s40359-023-01263-7 (PMC10422823; doi:10.1186/s40359-023-01263-7)
Supplement: Supplementary file 1 — Additional file 1. [file 40359_2023_1263_MOESM1_ESM.docx]

Questionnaire 1

for the research project

Mental Health and the Post-Covid Workplace

Head of research/contact: Isabella Mader [isabella.mader@excellence-research.at](mailto:isabella.mader@excellence-research.at)

Research institution: Excellence Research, Vienna

*Instructions for completion:
Please read each statement and mark the number 0, 1, 2, or 3 to indicate how much the statement applied to you* ***during the past week****. We will ask about experiences further back in Part 2 of the questionnaire below.*

*There are no right or wrong answers. Try to decide on an answer spontaneously.*

*0 ... Did not apply to me* ***at all****1 ... Applied to me* ***to some degree****, or* ***some of the time*** *2 ... Applied to me* ***to a considerable degree*** *or* ***a good part of time*** *3 ... Applied to me* ***very much*** *or* ***most of the time***

1. Questions about your well-being

| 1 | I found it difficult to calm down. | 0 | 1 | 2 | 3 |
| --- | --- | --- | --- | --- | --- |
| 2 | I felt that my mouth was dry. | 0 | 1 | 2 | 3 |
| 3 | I couldn't experience any positive emotions at all. | 0 | 1 | 2 | 3 |
| 4 | I had breathing problems (e.g. excessively fast breathing, shortness of breath without physical exertion). | 0 | 1 | 2 | 3 |
| 5 | It was difficult for me to motivate myself to get things done. | 0 | 1 | 2 | 3 |
| 6 | I tended to overreact to situations. | 0 | 1 | 2 | 3 |
| 7 | I trembled (e.g. in my hands). | 0 | 1 | 2 | 3 |
| 8 | I found everything exhausting. | 0 | 1 | 2 | 3 |
| 9 | I worried about situations in which I could panic and make a fool of myself. | 0 | 1 | 2 | 3 |
| 10 | I felt like I couldn't look forward to anything anymore. | 0 | 1 | 2 | 3 |
| 11 | I noticed that I became easily agitated. | 0 | 1 | 2 | 3 |
| 12 | I found it difficult to relax. | 0 | 1 | 2 | 3 |
| 13 | I felt down and sad. | 0 | 1 | 2 | 3 |
| 14 | I reacted angrily to anything that prevented me from continuing my current activity. | 0 | 1 | 2 | 3 |
| 15 | I felt close to a panic attack. | 0 | 1 | 2 | 3 |
| 16 | I was unable to get excited about anything. | 0 | 1 | 2 | 3 |
| 17 | I didn't feel like I was worth much as a person. | 0 | 1 | 2 | 3 |
| 18 | I found myself quite sensitive. | 0 | 1 | 2 | 3 |
| 19 | I felt my heartbeat without having physically exerted myself (e.g. feeling of palpitations or skipped beats). | 0 | 1 | 2 | 3 |
| 20 | I felt anxious for no reason. | 0 | 1 | 2 | 3 |
| 21 | I felt that life was meaningless. | 0 | 1 | 2 | 3 |

*Questions 1-21 are derived from the standardized DASS-21 questionnaire (Depression-Anxiety-Stress Scales).*

1. Additional questions

| 22 | I am currently undergoing treatment for the following diagnoses: |
| --- | --- |
| 23 | I am currently taking the following medications and/or supplements: |
| 24 | I have the following allergies/intolerances: |
| 25 | If you are currently not feeling well, please briefly describe in your own words how this manifests: |
| 26 | How long have you been feeling this way? (only if you are currently not feeling well, otherwise please continue with question 28) |
| 27 | Was there a trigger? (only if you are currently not feeling well, otherwise please continue with question 28): |
| 28 | Do you have any general medical conditions, especially such as diabetes, high blood pressure, etc.? |
| 29 | What are you currently doing for your well-being? Exercise? Special diet? Something else? Since when? What works well? |
| 30 | Do you meet many or few people in your daily life - professionally or privately? How was that (different?) under Covid? Less/more stress? Less pleasant? Or more pleasant? |
| 31 | What else do you think is important information - or what else would you like to tell us? |

Thank you very much!

For any inquiries, you can reach us at: [Isabella.mader@excellence-research.at](mailto:Isabella.mader@excellence-research.at)

__________________________ __________________________ __________________________

**Date Case number Age**

*(please do not enter your name,
please only use the case number
for reasons of anonymity)*
