# Supplementary material for: Recovery from (treatment-resistant) depression after lifestyle changes and micronutrient precision supplementation: a preliminary field study in patients
Source: BMC Psychol. 2023 Aug 11;11:229. doi: 10.1186/s40359-023-01263-7 (PMC10422823; doi:10.1186/s40359-023-01263-7)
Supplement: Supplementary file 2 — Additional file 2. [file 40359_2023_1263_MOESM2_ESM.docx]

Questionnaire 2

for the research project

Mental Health and the Post-Covid Workplace

Head of research/contact: Isabella Mader [isabella.mader@excellence-research.at](mailto:isabella.mader@excellence-research.at)

Research institution: Excellence Research, Vienna

*Instructions for completion:
Please read each statement and mark the number 0, 1, 2, or 3 to indicate how much the statement applied to you* ***during the past week****. We will ask about experiences further back in Part 2 of the questionnaire below.*

*There are no right or wrong answers. Try to decide on an answer spontaneously.*

*0 ... Did not apply to me* ***at all****1 ... Applied to me* ***to some degree****, or* ***some of the time*** *2 ... Applied to me* ***to a considerable degree*** *or* ***a good part of time*** *3 ... Applied to me* ***very much*** *or* ***most of the time***

1. Questions about your well-being

| 1 | I found it difficult to calm down. | 0 | 1 | 2 | 3 |
| --- | --- | --- | --- | --- | --- |
| 2 | I felt that my mouth was dry. | 0 | 1 | 2 | 3 |
| 3 | I couldn't experience any positive emotions at all. | 0 | 1 | 2 | 3 |
| 4 | I had breathing problems (e.g. excessively fast breathing, shortness of breath without physical exertion). | 0 | 1 | 2 | 3 |
| 5 | It was difficult for me to motivate myself to get things done. | 0 | 1 | 2 | 3 |
| 6 | I tended to overreact to situations. | 0 | 1 | 2 | 3 |
| 7 | I trembled (e.g. in my hands). | 0 | 1 | 2 | 3 |
| 8 | I found everything exhausting. | 0 | 1 | 2 | 3 |
| 9 | I worried about situations in which I could panic and make a fool of myself. | 0 | 1 | 2 | 3 |
| 10 | I felt like I couldn't look forward to anything anymore. | 0 | 1 | 2 | 3 |
| 11 | I noticed that I became easily agitated. | 0 | 1 | 2 | 3 |
| 12 | I found it difficult to relax. | 0 | 1 | 2 | 3 |
| 13 | I felt down and sad. | 0 | 1 | 2 | 3 |
| 14 | I reacted angrily to anything that prevented me from continuing my current activity. | 0 | 1 | 2 | 3 |
| 15 | I felt close to a panic attack. | 0 | 1 | 2 | 3 |
| 16 | I was unable to get excited about anything. | 0 | 1 | 2 | 3 |
| 17 | I didn't feel like I was worth much as a person. | 0 | 1 | 2 | 3 |
| 18 | I found myself quite sensitive. | 0 | 1 | 2 | 3 |
| 19 | I felt my heartbeat without having physically exerted myself (e.g. feeling of palpitations or skipped beats). | 0 | 1 | 2 | 3 |
| 20 | I felt anxious for no reason. | 0 | 1 | 2 | 3 |
| 21 | I felt that life was meaningless. | 0 | 1 | 2 | 3 |

*Questions 1-21 are derived from the standardized DASS-21 questionnaire (Depression-Anxiety-Stress Scales).*

1. Additional questions

| 22 | Please describe briefly in your own words how you are currently feeling. |
| --- | --- |
| 23 | Have there been any changes in other diagnoses? (if applicable) |
| 24 | In the last 2 months, I have taken the recommended vitamins (click to check/select)  consistently  modified: (how?)  stopped: (why?)  other: (please specify if possible) |
| 25 | In the last 2 months, I have followed the following behavioral recommendations:  socialized more  exercise (please tell us what type): _  avoided bright light/phone/TV at night  ate fresh foods, avoided heavily processed foods  How successful was it? ____  Comments: |
| 26 | Which micronutrients/vitamins and behavioral recommendations will you continue to follow? |
| 27 | Were the study materials useful, understandable, and interesting? If you would like to provide feedback, we would be very grateful. |
| 28 | May we contact you again in a few months to ask how you are doing? |
| 29 | What else do you think is important information to provide or anything else you would like to tell us? |

Thank you very much!

For any inquiries, you can reach us at: [Isabella.mader@excellence-research.at](mailto:Isabella.mader@excellence-research.at)

__________________________ __________________________

**Date Case number**

*(for reasons of anonymity, please do not enter your name)*
